# Supplementary figures and images for: Lipid exchange at ER–trans-Golgi contact sites governs polarized cargo sorting
Source: J Cell Biol. 2023 Nov 22;223(1):e202307051. doi: 10.1083/jcb.202307051 (PMC10664280; doi:10.1083/jcb.202307051)

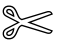

**Upper part: Anti-OSBP**

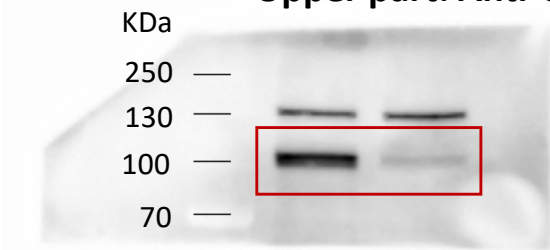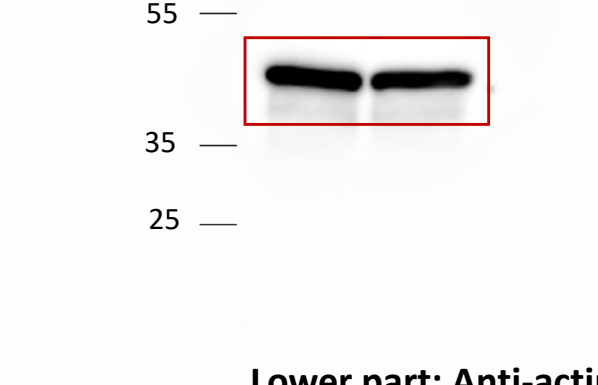

**Lower part: Anti-actin**

Supplement: SourceData F1 — is the source file for Fig. 1. [file JCB_202307051_SourceDataF1.pdf]

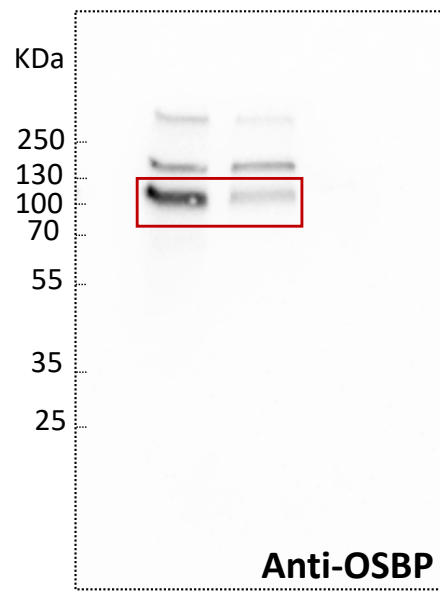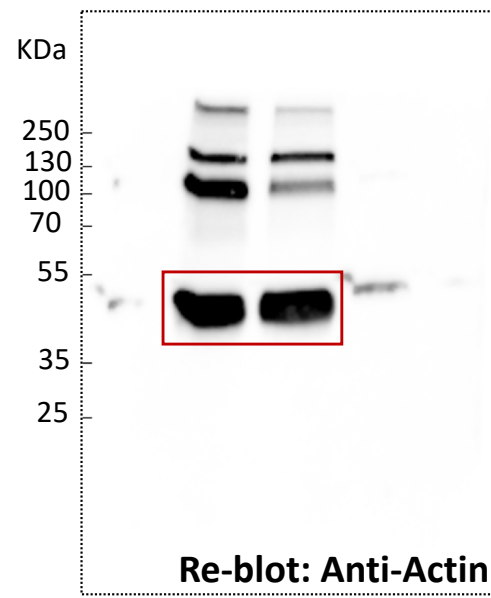

Supplement: SourceData FS1 — is the source file for Fig. S1. [file JCB_202307051_SourceDataFS1.pdf]

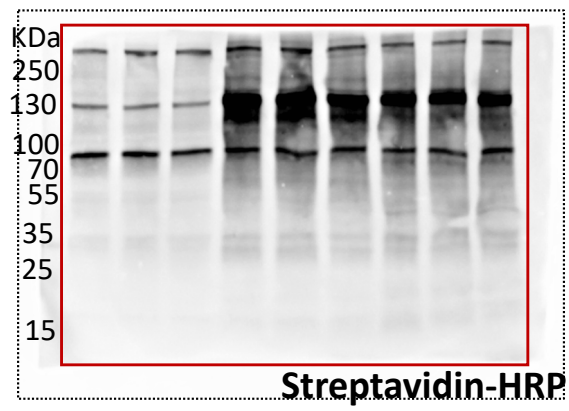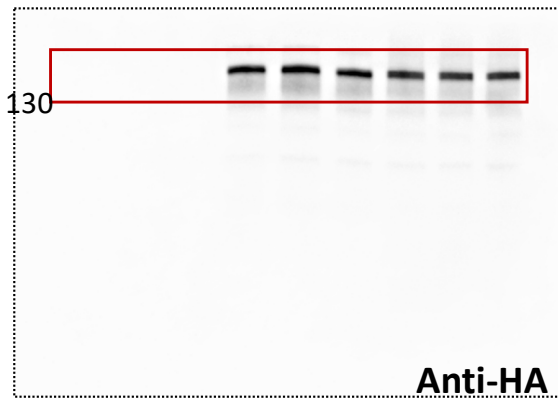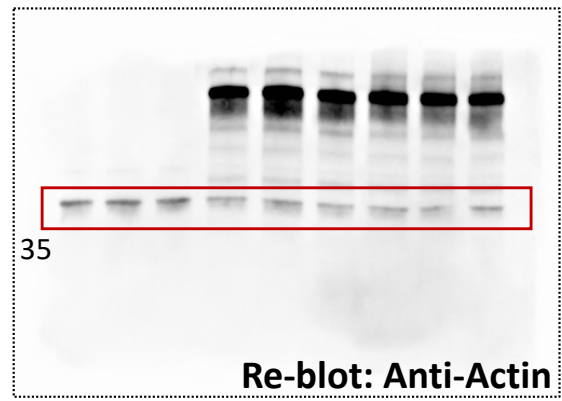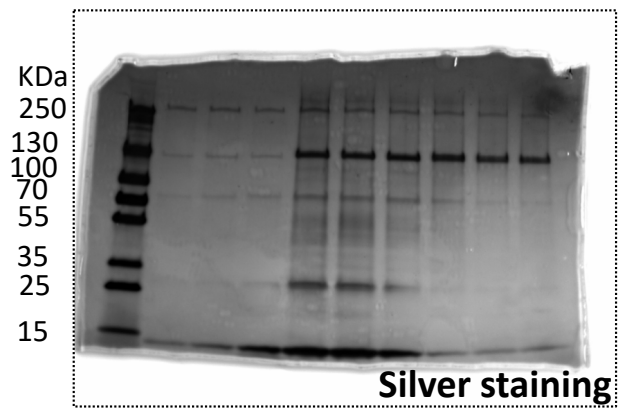

Supplement: SourceData FS3 — is the source file for Fig. S3. [file JCB_202307051_SourceDataFS3.pdf]
